# Socioeconomic inequalities in newborn care during facility and home deliveries: a cross sectional analysis of data from demographic surveillance sites in rural Bangladesh, India and Nepal

**DOI:** 10.1186/s12939-018-0834-9

**Published:** 2018-08-15

**Authors:** Erik de Jonge, Kishwar Azad, Munir Hossen, Abdul Kuddus, Dharma S. Manandhar, Ellen van de Poel, Swati Sarbani Roy, Naomi Saville, Aman Sen, Catherine Sikorski, Prasanta Tripathy, Anthony Costello, Tanja A. J. Houweling

**Affiliations:** 1 Department of Public Health, Erasmus University Medical Center, P.O. Box 2040, 3000 CA Rotterdam, the Netherlands; 2 Perinatal Care Project, Diabetic Association of Bangladesh, 122 KaziNazrul Islam Avenue, Dhaka, 1000 Bangladesh; 3 Mother Infant Research Activities (MIRA), YB Bhavan, Thapathali, Kathmandu, 921 Nepal; 4 Ekjut, Plot 556B, Potka, Chakradharpur, West Singhbhum, Jharkhand India; 5 Institute of Health Policy and Management, Erasmus University Rotterdam, Rotterdam, the Netherlands; 6 Institute for Global Health, University College London, London, UK

## Abstract

**Background:**

In Bangladesh, India and Nepal, neonatal outcomes of poor infants are considerably worse than those of better-off infants. Understanding how these inequalities vary by country and place of delivery (home or facility) will allow targeting of interventions to those who need them most. We describe socio-economic inequalities in newborn care in rural areas of Bangladesh, Nepal and India for all deliveries and by place of delivery.

**Methods:**

We used data from surveillance sites in Bangladesh, India and from Makwanpur and Dhanusha districts in Nepal, covering periods from 2001 to 2011. We used literacy (ability to read a short text) as indicator of socioeconomic status. We developed a composite score of nine newborn care practices (score range 0–9 indicating infants received no newborn care to all nine newborn care practices). We modeled the effect of literacy and place of delivery on the newborn care score and on individual practices.

**Results:**

In all study sites (60,078 deliveries in total), use of facility delivery was higher among literate mothers. In all sites, inequalities in newborn care were observed: the difference in new born care between literate and illiterate ranged 0.35–0.80. The effect of literacy on the newborn care score reduced after adjusting for place of delivery (range score difference literate-illiterate: 0.21–0.43).

**Conclusion:**

Socioeconomic inequalities in facility care greatly contribute to inequalities in newborn care. Improving newborn care during home deliveries and improving access to facility care are a priority for addressing inequalities in newborn care and newborn mortality.

**Electronic supplementary material:**

The online version of this article (10.1186/s12939-018-0834-9) contains supplementary material, which is available to authorized users.

## Background

Neonatal mortality in Bangladesh, India and Nepal is high and socioeconomic inequalities in neonatal outcomes are substantial [1, 2]. Although there is agreement about the interventions that would save most newborn lives, [3, 4] there are considerable socioeconomic inequalities in coverage of such newborn care interventions [5, 6].

Whether newborn care interventions should be delivered at home or in the facility is an important question, both in terms of quality of care and from an equity point of view. Newborn care is much better when a delivery takes place in a health facility, [7] but use of health facility delivery is highly unequal [8]. To what extent can inequalities in newborn care be explained by place of delivery?

Much of the research on inequalities in reproductive, maternal, newborn and child care relies on the Demographic Health Surveys (DHS) [9]. The DHSs are nationally-representative household surveys that provide data for monitoring indicators in population, health, and nutrition. Funded by USAID and other donors, the DHS surveys are carried out in more than 90 developing countries. Bangladesh has implemented six surveys since 1993, Nepal six surveys since 1987 and India three surveys since 1992. In 2007 the DHS in Bangladesh began to collect data on newborn care with questions on the use of clean instruments to cut the umbilical cord, cord care, bathing delays and prevention of hypothermia [10]. In 2011, the DHS in Nepal began to collect similar data on newborn care, underscoring the importance of measuring improvements in newborn care practices to improve neonatal survival [11]. However, the data on newborn care were only collected for women giving birth at home, which makes it impossible to compare home and facility deliveries. Furthermore, the DHS newborn care questions were asked to women who gave birth up to three years ago, which may result in recall bias.

In this paper we describe and compare socio-economic inequalities in newborn care by place of delivery using data from four demographic surveillance sites in rural areas of Bangladesh, Nepal and India in order to guide policies for reducing inequalities in coverage of these practices and in neonatal mortality.

## Methods

### Study population

We analyzed data from demographic surveillance sites, located in Jharkhand and Odisha state in India (*n* = 8720), Dhanusha district (*n* = 17,835) and Makwanpur district (*n* = 6688) in Nepal and Bogra, Faridpur and Moulvibazar districts in Bangladesh (*n* = 26,835). The surveillance sites were developed for cluster-randomized trials of a community intervention to reduce maternal and neonatal mortality. Table 1 provides details on the sites. In this study, we analyzed data from the control arm of the trials, because the intervention is known to have affected newborn care practices [12]. We included all live born infants. If a mother delivered twins or triplets, we only included the infant born first.

**Table 1:** Brief descriptions of the demographic surveillance sites

	Bangladesh	India	Nepal (Makwanpur)	Nepal (Dhanusha)
Location	Bogra, Maulvibazaar and Faridpur districts	West Singhbhum and Saraikela Districts (Jharkhand); Keonjhar Districts (Odisha)	Makwanpur district, central region mid-hills	Dhanusha district, central plains of Nepal
Period	February 1st 2005–31 December 2009	1st July 2005 - 30th June 2008	1st November 2001 - 31st October 2004 (phase 1)1st November 2004 - 31st October 2008 (phase 2)	1st September 2006 - 13th April 2011
Number of clusters (number included in the study)	18(9)	36(18)	24(12) (Phase 1) 30(6) (Phase 2 -former control clusters became intervention clusters and 6 new control clusters recruited)	60(30)
Annual births sampled per cluster (number included in the study)	596 (119)	171(38)	115(70)	104(17)
Approximate cluster population	28,000	6400	4000	8000

### Data collection

Surveillance of all births, neonatal and maternal deaths in the study areas was done by key informants. Key informants were usually traditional birth attendants in Bangladesh and incentivized ‘enumerators’ in Nepal and India, recruited to cover approximately 250 households each. The key informant notified a salaried interviewer when a mother delivered or when a death occurred. The interviewer would then visit the mother at home to conduct a structured interview around 6 weeks after delivery. The interview addressed the outcome of the pregnancy, home care practices and health care-seeking during pregnancy, delivery and the neonatal period, as well as socioeconomic and demographic information like educational attainment and household assets. Data were collected on paper, checked by auditors, entered by separate data entry operators, and cross-checked by data managers for data quality purposes.

### Outcomes: Newborn care practices

In order to compare newborn care at home and in health facilities, we selected a set of newborn care practices that should be performed during or after any delivery, both at home and in the facility, following guidance from the WHO on essential newborn care (Table 2) [13].

**Table 2:** Newborn care practices included in the essential newborn care score for each study site

Hand washing	Birth attendant washed hands before delivery
Clean delivery kit	A clean delivery kit (CDK) was used during the delivery. A CDK usually contains a small bar of soap for washing hands, a plastic sheet to serve as the delivery surface, clean string for tying the umbilical cord, a new razor blade for cutting the cord, and pictorial instructions that illustrate the sequence of delivery events and hand-washing.
Clean instrument	The umbilical cord was cut with a sterilized instrument (new or boiled razor blade, surgical blade or scissors).
Appropriate cord care	After cutting the cord either dry cord care was practised or an antiseptic was applied to the stump.
Wrapped within 5 mins	The baby was wrapped in clean cloth within 5 mins after delivery or placed skin-to-skin on the breast of the mother.
Bathing after 6 h	Bathing of the baby was delayed until at least 6 h after the delivery
Breast feeding within 1 h	The mother initiated breastfeeding within the first hour of delivery.
No prelacteal feeding	The baby was exclusively fed with breast milk during the first 24 h of life and not fed any other fluid or prelacteal food.
Postnatal care	The mother and her baby were seen for a postnatal check-up by a health care worker (doctor, nurse or nurse-midwife) 24 h after delivery

In order to summarize newborn care practices for the analysis, a score was calculated by summing these indicators. All indicators were coded as 1 if a practice was conducted per WHO guidance on newborn care and 0 if not, which resulted in a newborn care score ranging from 0 to 9. All care practices we allocated equal importance so no weights were applied.

### Predictors: Literacy and place of delivery

Literacy and place of delivery were predictors in our analysis. Literacy was defined as the ability to read a short text during the interview and used as indicator of socio-economic status. Place of delivery had three categories: health facility (hospital or health center) or at home with a skilled birth attendant or “SBA” (a doctor, a nurse, a government health worker, or an auxiliary nurse midwife)(home+SBA) or at home without an SBA (home-SBA).

### Missing data

A considerable number of observations had missing values for the practices, due to changes in skip patterns in, and differing versions of, the questionnaires over time. In Nepal (Dhanusha) 53%, India 17%, Nepal (Makwanpur) 73%, Bangladesh 30% of all observations had one or more of the practices missing which meant that the total score was missing as it is a sum of nine newborn care practices. Missing values were higher for hospital deliveries (Nepal (Dhanusha) 88% of observations had at least one care practice missing, India 46%, Nepal (Makwanpur) 69%, Bangladesh 75%). The care practice with most missing observations was hand washing for Nepal (Dhanusha) (44%) and Bangladesh (22%) and clean instrument to cut the cord for India (12%) and Nepal (Makwanpur) (45%). Additional file 1 provides a detailed breakdown of the missing values.

Missing data were imputed, replacing missing data with estimated data. This is preferable over analyzing complete cases only, because by removing incomplete cases one loses power and, more importantly, there might be patterns of missingness that would confound results in a complete case analysis. If the available data explains missingness, analysis of imputed data will be less confounded. There should be no systematic explanations for missingness outside the explanatory variables in the imputation model though, otherwise the results will still be confounded. This cannot be formally tested, but one can test the robustness of the results against an analysis of complete cases, which we did.

We used multiple imputation by chained equations (MICE) [14]. Perfect prediction was corrected using an augmented-regression approach [15]. Geographical cluster was included as independent variable in the imputation model to adjust for the clustered sampling design of the demographic surveillance sites. Five imputed datasets were created for estimation of the models.

### Statistical analysis

We analyzed the effect of literacy on the newborn care score in a univariable analysis and the effect of literacy and place of delivery in a multivariable analysis. An interaction term of literacy and place of delivery was included in the second model to study possible differences in the effect of literacy by place of delivery. The analyses were done using a random effect models to adjust for clustering.

Analyses were done using STATA, version 13.

### Ethical approval

All trials that provided underlying data for this study were approved by the ethics committee of the Institute of Child Health and Great Ormond Street Hospital for Children (UK) and by the following research ethics committees: the ethical review committee of the Diabetic Association of Bangladesh; an independent ethics committee in Jamshedpur, India (Eastern India trial); the Nepal Health Research Council (Dhanusha and Makwanpur, Nepal). All trials were conducted in disadvantaged areas with high levels of female illiteracy; all participants gave consent in writing, by thumbprint or verbally.

## Results

Most women in the Nepal and India sites were illiterate (over two-thirds), compared with only one third of women in the Bangladesh site (Table 3). A minority of women in all sites delivered in a facility (2% of women in Nepal (Makwanpur); around 20% in the other sites). Skilled birth attendance during a home delivery was rare (0 to3%). Uptake of facility delivery was substantially higher among literate women than among illiterate women.

**Table 3:** The distribution of literacy and delivery type in the population of the study sites

	India	Bangladesh	Nepal (Dhanusha)	Nepal (Makwanpur)
Number of deliveries	8720	26,835	17,835	6688
Literacy				
Illiterate (%)	68%	34%	76%	66%
Literate (%)	32%	66%	24%	34%
Place of delivery				
Home – SBA (%)	78%	80%	78%	97%
Home + SBA (%)	3%	3%	1%	0%
Facility (%)	19%	17%	21%	2%
Delivery type by literacy				
Literate				
Home – SBA (%)	61%	76%	64%	94%
Home + SBA (%)	5%	3%	1%	1%
Facility (%)	34%	21%	35%	5%
Illiterate				
Home – SBA (%)	86%	89%	83%	99%
Home + SBA (%)	3%	2%	1%	0%
Facility (%)	11%	8%	16%	1%

**Fig. 1:**
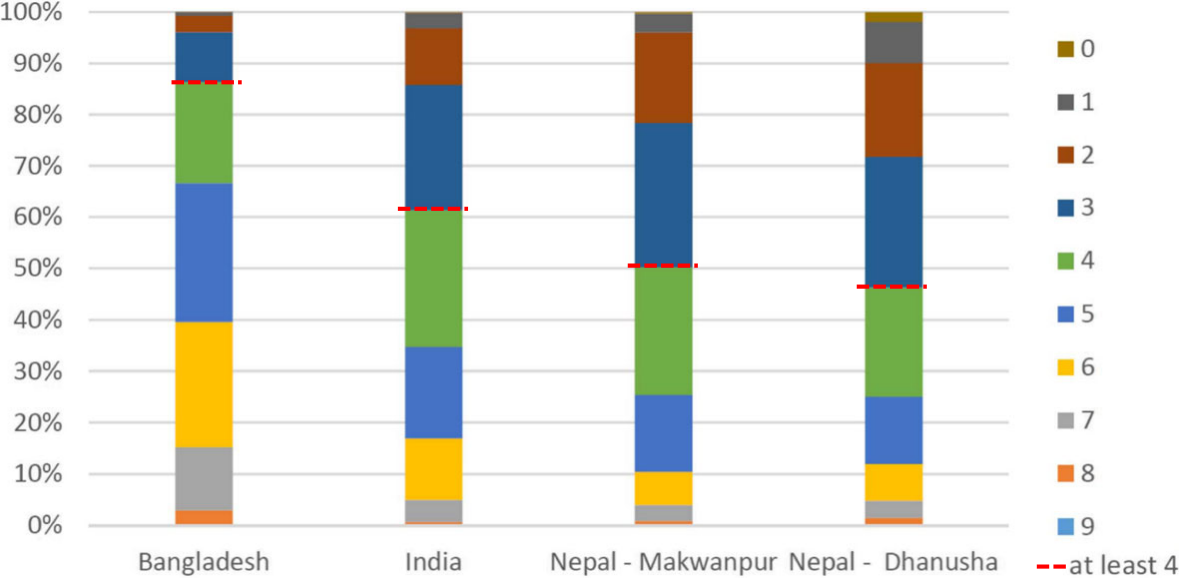
The proportion of deliveries by number of newborn care practices conducted during the delivery. The red dotted line indicates the proportion of deliveries per site with at least four newborn care practices

Uptake of newborn care practices varied strongly between individual practices (e.g. postnatal care was rare, while not giving prelacteal foods was fairly common in all sites), but was far from universal for most practices (Table 4). Uptake also varied strongly between sites. While for most (86%) deliveries in Bangladesh at least four newborn practices were done; this was the case for less than half (46%) of deliveries in Nepal (Dhanusha) (Fig. 1).

**Table 4:**
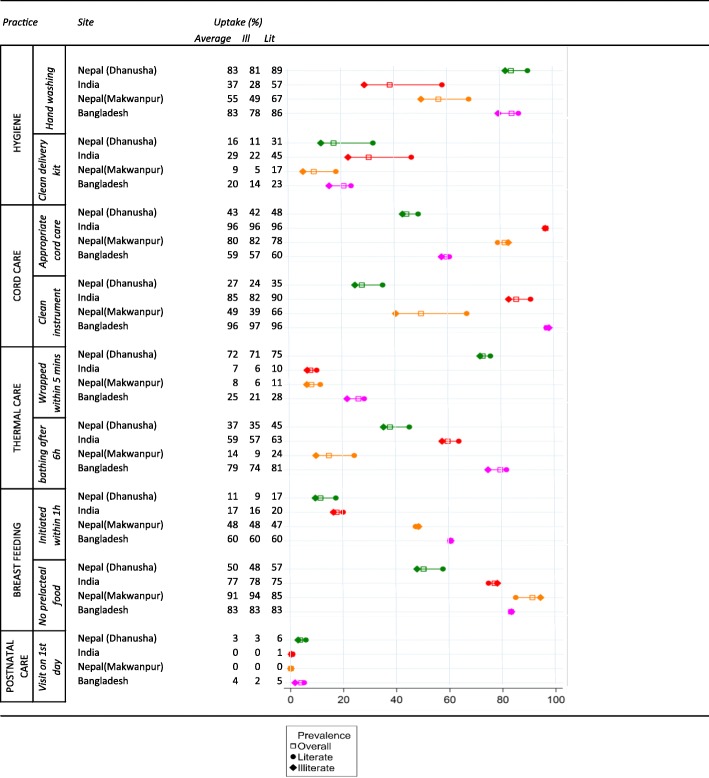
Uptake of each individual newborn care practice for the entire population and by literacy (%)

Infants of literate mothers were more likely to receive the newborn care practices than infants of illiterate mothers, with a few exceptions (e.g. no prelacteal feeding) (Table 4). A large gap in newborn care practices was observed between illiterate women delivering at home without a SBA and literate women delivering in a facility. In a given place of birth, socioeconomic inequalities in newborn care were, in absolute terms, usually fairly modest (Table 5).

**Table 5:**
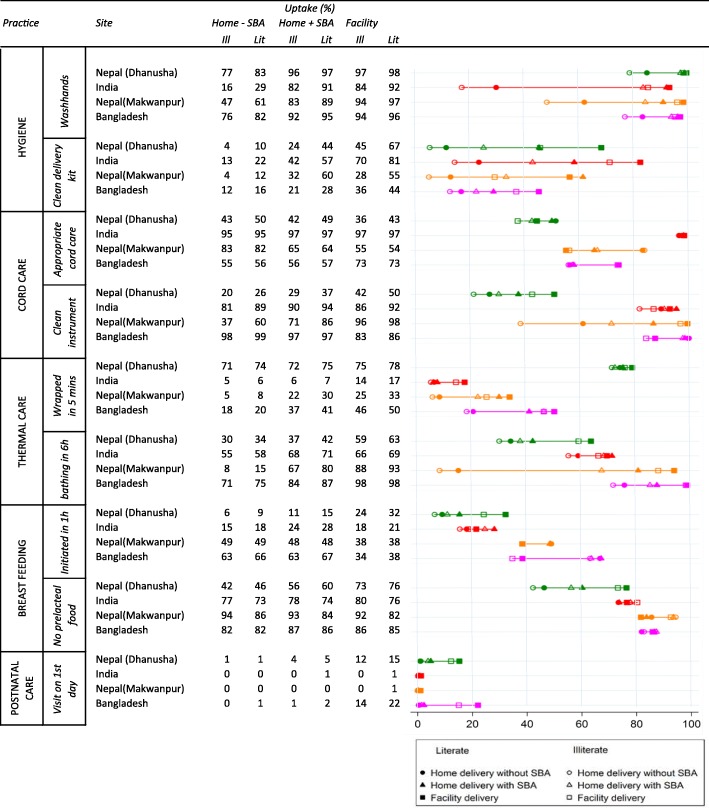
Uptake of individual newborn care practices by place of delivery and literacy (%)

Socioeconomic inequalities in newborn care were also reflected in the higher newborn care score for deliveries among literate mothers compared with illiterate mothers (difference in newborn care score ranging from 0.35 (0.31, 0.39) in Bangladesh to 0.80 (0.73, 0.87) in Nepal (Dhanusha) on a scale of 0 to 9) (Table 6). These socioeconomic inequalities in the newborn care score, as indicated by the beta, became considerably smaller - now ranging from 0.21 (95% CI: 0.17–0.25) in Bangladesh to 0.43 (95% CI: 0.36–0.51) in Nepal (Makwanpur) - in a multivariable analysis that also included place of delivery and an interaction term for place of delivery * literacy. In this analysis, the differences in newborn care between literate and illiterate women were much smaller than differences in the newborn care score between facility deliveries and home deliveries without SBA, which ranged from 1.00 (95% CI: 0.90–1.11 (Bangladesh) to 2.08 (95% CI: 1.83–2.32) (Nepal (Makwanpur)) units of the score. Socioeconomic inequalities in newborn care score were usually of similar magnitude at home with or without an SBA and in the facility, as indicated by the small and statistically insignificant interaction terms between literacy and place of delivery in all sites, except Nepal (Makwanpur).

**Table 6:** A model of the effect of literacy on newborn care score (model) and the same model with place of delivery as additional predictor and an interaction term of literacy* place of delivery (model 2)

	India	Bangladesh	Nepal (Dhanusha)	Nepal (Makwanpur)
	beta (95% CI)	beta (95% CI)	beta (95% CI)	beta (95% CI)
Model 1				
Illiterate	ref	ref	ref	ref
Literate	0.66 (0.59–0.72)	0.35 (0.31–0.39)	0.80 (0.73–0.87)	0.65 (0.57–0.72)
Constant	3.85 (3.60–4.11)	4.86 (4.60–5.12)	3.27 (3.13–3.41)	3.37 (3.23–3.52)
Model 2				
Illiterate	ref	ref	ref	ref
Literate	0.28 (0.22–0.35)	0.21 (0.17–0.25)	0.42 (0.35–0.48)	0.43 (0.36–0.51)
Home - SBA	ref	ref	ref	ref
Home+SBA	1.12 (0.94–1.30)	0.61 (0.46–0.75)	0.83 (0.60–1.06)	1.22 (0.61–1.82)
Facility	1.42 (1.32–1.51)	1.00 (0.90–1.11)	1.77 (1.66–1.87)	2.08 (1.83–2.32)
Home + SBA*literate	0.15 (− 0.11–0.41)	0.04 (− 0.13–0.22)	0.08 (− 0.29–0.44)	0.69 (− 0.03–1.41)
Facility*literate	0.04 (− 0.09–0.17)	−0.02 (0.14–0.09)	0.11 (− 0.02–0.25)	− 0.33 (0.62 - -0.05)
Constant	3.61 (3.39–3.84)	4.74 (4.45–5.04)	2.96 (2.82–3.10)	3.30 (3.19–3.41)

## Discussion

We found that newborn care is better in higher socioeconomic groups than in lower socioeconomic groups in rural areas of Bangladesh, India and Nepal. These inequalities are to a large extent explained by the higher percentage of facility deliveries among literate women compared with illiterate women and better newborn care practices in facilities. Infants of illiterate mothers receive more appropriate care in a facility than infants of literate mothers at home. Nevertheless, inequalities in newborn care also exist in facilities with illiterate mothers receiving poorer care.

Our study has some limitations. Firstly, a considerable number of observations had missing information on newborn care practices, especially for women delivering in a facility due to a skip pattern in one version of the questionnaire. We solved the missing data problem through imputation. We performed a complete case analysis for comparison with the analysis on the imputed data. The analysis gave comparable results, suggesting that, conditional on the variables included in the imputation model, missingness was at random (Additional file 2).

A second limitation is that the data were collected at least 6 weeks after delivery, which may have resulted in recall bias. However, recall bias in our data is likely to be much lower than in the DHS, which use a recall period of up to 2 years. Finally, the studies were collected over differing time periods (see Table 1), so we do not have concurrent comparisons of the uptake of newborn care practices by site.

A third limitation is that all trials were conducted in disadvantaged areas with high levels of female illiteracy, which should be taken into account when generalizing the results of the analysis to other areas.

In our data we had access to several potential SES indicators: literacy, maternal education and assets. We selected literacy because it is commonly used in health research in developing countries [16–18], it allowed us to categorize low SES in a simple way and was collected consistently across sites. Maternal education in years was available but in different categories across sites. We considered calculating an asset index, which is another indicator often used in health research from developing countries [19]. In our study limited numbers of different assets were collected for the different sites. Generally, calculating asset indices using different sets of assets results in different definitions of low SES [20], so using different sets between sites to calculate the indices in our study might have resulted in an inconsistent definition of low SES.

Improving newborn care for every newborn contributes to reducing neonatal mortality, so addressing inequalities in newborn care can arguably contribute to reduced neonatal mortality inequalities [4, 21]. Addressing inequalities in newborn care requires a two-pronged strategy: improving access to good quality facility delivery care, especially for poor women, while at the same time improving practices for home births.

Our findings support the strong push in the international literature promoting facility delivery in low and middle income countries [4, 22]. The differences in newborn care between facility and home delivery are much larger than between illiterate and literate women. Also among illiterate women, newborn care is much better in the facility than at home. Our analysis only looked at care practices that should happen to every newborn, so the added value of ‘higher level’ comprehensive emergency obstetric and neonatal care was not taken into account.

Steps have been made in Bangladesh, India and Nepal to increase supply of facility delivery care, although progress is limited by human resources [23, 24]. Large inequalities in uptake of facility delivery, as well as the more general understanding that improved access to medical technology first benefits the better-off, suggest that policies to increase access should include some form of targeting [8, 25]. Demand side strategies, like conditional cash transfer schemes, have been put in place in India, Bangladesh and Nepal, to allow women of lower socioeconomic status to access facility delivery care [23, 24]. Evidence suggests that these schemes improve access to, and reduce inequalities in, use of facility delivery care, which should improve inequalities in newborn care as well [26].

Inequality in coverage of newborn care practices between literate and illiterate populations delivering in health facilities is a cause for concern. Ideally, once reaching care in a health facility, quality of care should be equivalent for everyone regardless of socioeconomic position. The observed differences can be caused by differences in health facilities with the literate mothers using facilities with a higher standard of care, or due to health workers differentiating their behavior on the basis of the patients’ literacy status. Further research is needed to tease this out. Low coverage of some practices even among literate women delivering in in facilities (e.g. postnatal care) is another cause for concern and suggests that there is ample room for improvement in quality of care during facility deliveries.

Efforts to improve quality of home deliveries will also contribute to equity in newborn care. Community-based interventions including community support groups and home visits were associated with increased use of clean delivery kits for home births and early initiation of breastfeeding [27]. Trials in India, Nepal and Bangladesh have demonstrated that participatory learning and action through women’s groups can improve newborn care practices in home deliveries across all socioeconomic strata [12, 28, 29]. Given that most deliveries in low- and middle income countries still take place at home, these interventions should be included in national newborn care policies.

In order to ensure that newborn care policies are improving access for the poor, their equity effects must be monitored over time. Crowe, et al. analyzed the same demographic surveillance data as we did and showed that the quality of newborn care during attended deliveries reduced over time. For example, rates of hygienic cord-cutting and skin-to-skin contact during attended deliveries fell in Bangladesh over the period 2005–2009, while rates of attended delivery increased [30]. One would be interested to know the equity trend, hypothesizing that the reduced quality of newborn care during facility deliveries would be observed in mainly poor mothers, resulting in bigger inequality in newborn care. Barros et al. developed a framework to monitor equity trends in coverage of maternal, neonatal and child health, but this framework was developed for analyzing DHS datasets and only includes “skilled birth attendance”, without looking at individual newborn care practices or distinguishing between place of delivery [31]. We recommend that monitoring equity of newborn care includes place of delivery and that the Demographic Health Surveys should be expanded to include newborn care practices for facility deliveries as well as for home births.

## Conclusions

In summary, socioeconomic status strongly influences newborn care. Within a given place of delivery, socioeconomic status is less important for the newborn care received. Newborn care is much better in health facilities than at home, even for illiterate women, which is an argument for improving access to facility delivery care and improving newborn care practices in the home setting.

## Additional files

Additional file 1: **Table S1.** Missing data by newborn care practice. **Table S2.** Missing data by newborn care practice for home deliveries without a skilled birth attendant. **Table S3.** Missing data by newborn care practice for home deliveries with a skilled birth attendant. **Table S4.** Missing data by newborn care practice for facility deliveries. (PDF 41 kb)

Additional file 2: **Table S5.** Complete case analysis: The distribution (%) of literacy, delivery type and delivery type by literacy in the population of the study sites. **Table S6.** Complete case analysis: A univariable model of the effect of literacy on newborn care score. **Table S7.** Complete case analysis: A multivariable model of the effect of literacy and delivery type on newborn care score including interaction. (PDF 86 kb)
