# Supplementary material for: Socioeconomic inequalities in newborn care during facility and home deliveries: a cross sectional analysis of data from demographic surveillance sites in rural Bangladesh, India and Nepal
Source: Int J Equity Health. 2018 Aug 15;17:119. doi: 10.1186/s12939-018-0834-9 (PMC6094873; doi:10.1186/s12939-018-0834-9)
Supplement: Supplementary file 1 — Table S1. Missing data by newborn care practice. Table S2. Missing data by newborn care practice for home deliveries without a skilled birth attendant. Table S3. Missing data by newborn care practice for home deliveries with a skilled birth attendant. Table S4. Missing data by newborn care practice for facility deliveries. (PDF 41 kb) [file 12939_2018_834_MOESM1_ESM.pdf]

Appendix 1.

Table 1. Missing data by newborn care practice.

	Nepal(Dhanusha)		India		Nepal(Makwanpur)		Bangladesh	
Total number of observations	18,470		8,978		6,829		27,788	
Total number of missing observations (% of total observations)								
Essential Newborn Care (ENC) score (sum of 9 practices)	9,859	(53%)	1,565	(17%)	5,010	(73%)	8,345	(30%)
Hand washing by birth attendant (Washhands)	8,075	(44%)	646	(7%)	2,197	(32%)	6,028	(22%)
Clean delivery kit used (CDK)	5,098	(28%)	101	(1%)	2,010	(29%)	2,591	(9%)
Appropriate cord care (CordCare)	4,060	(22%)	262	(3%)	364	(5%)	1,612	(6%)
Clean instrument used to cut cord (CordCut)	3,782	(20%)	1,084	(12%)	3,069	(45%)	4,685	(17%)
Wrapped within 5 mins (BabyWarm)	3,683	(20%)	271	(3%)	0	(0%)	990	(4%)
Bathing after 6h (Bathing)	3,995	(22%)	313	(3%)	0	(0%)	1,104	(4%)
Breast feeding Initiated within 1h (Breast)	1,192	(6%)	286	(3%)	55	(1%)	1,052	(4%)
No prelacteal food (OnlyBreastMilk)	1,003	(5%)	261	(3%)	0	(0%)	976	(4%)
Postnatal visit on 1st day (PNCChild_Day)	160	(1%)	4	(0%)	0	(0%)	22	(0%)

Table 2 Missing data by newborn care practice for home deliveries without a skilled birth attendant

	Nepal(Dhanusha)		India		Nepal(Makwanpur)		Bangladesh	
Total number of observations	13,411		6,731		6,097		21,262	
Total number of missing observations (% of total observations)								
Essential Newborn Care (ENC) score (sum of 9 practices)	5,711	(43%)	599	(9%)	4,515	(74%)	3,967	(19%)
Hand washing by birth attendant (Washhands)	4,239	(32%)	336	(5%)	1,968	(32%)	2,794	(13%)
Clean delivery kit used (CDK)	1,537	(11%)	35	(1%)	1,694	(28%)	252	(1%)
Appropriate cord care (CordCare)	623	(5%)	152	(2%)	197	(3%)	949	(4%)
Clean instrument used to cut cord (CordCut)	396	(3%)	260	(4%)	2,658	(44%)	834	(4%)
Wrapped within 5 mins (BabyWarm)	468	(3%)	150	(2%)	0	(0%)	579	(3%)
Bathing after 6h (Bathing)	698	(5%)	157	(2%)	0	(0%)	665	(3%)
Breast feeding Initiated within 1h (Breast)	689	(5%)	167	(2%)	39	(1%)	615	(3%)
No prelacteal food (OnlyBreastMilk)	545	(4%)	150	(2%)	0	(0%)	588	(3%)
Postnatal visit on 1st day (PNCChild_Day)	89	(1%)	1	(0%)	0	(0%)	9	(0%)

Appendix 1

Table 3 Missing data by newborn care practice for home deliveries with a skilled birth attendant

	Nepal(Dhanusha)		India		Nepal(Makwanpur)		Bangladesh	
Total number of observations	334		377		59		1,097	
Total number of missing observations (% of total observations)								
Essential Newborn Care (ENC) score (sum of 9 practices)	138	(41%)	106	(28%)	29	(49%)	367	(33%)
Hand washing by birth attendant (Washhands)	88	(26%)	40	(11%)	8	(14%)	185	(17%)
Clean delivery kit used (CDK)	66	(20%)	6	(2%)	17	(29%)	85	(8%)
Appropriate cord care (CordCare)	35	(10%)	30	(8%)	11	(19%)	81	(7%)
Clean instrument used to cut cord (CordCut)	26	(8%)	75	(20%)	17	(29%)	186	(17%)
Wrapped within 5 mins (BabyWarm)	25	(7%)	31	(8%)	0	(0%)	39	(4%)
Bathing after 6h (Bathing)	38	(11%)	32	(8%)	0	(0%)	45	(4%)
Breast feeding Initiated within 1h (Breast)	26	(8%)	31	(8%)	2	(3%)	43	(4%)
No prelacteal food (OnlyBreastMilk)	29	(9%)	30	(8%)	0	(0%)	42	(4%)
Postnatal visit on 1st day (PNCChild_Day)	8	(2%)	0	(0%)	0	(0%)	2	(0%)

Table 4 Missing data by newborn care practice for facility deliveries

	Nepal(Dhanusha)		India		Nepal(Makwanpur)		Bangladesh	
Total number of observations	4,234		1,831		672		5,194	
Total number of missing observations (% of total observations)								
Essential Newborn Care (ENC) score (sum of 9 practices)	3,705	(88%)	850	(46%)	465	(69%)	3,920	(75%)
Hand washing by birth attendant (Washhands)	3,530	(83%)	267	(15%)	220	(33%)	3,000	(58%)
Clean delivery kit used (CDK)	3,338	(79%)	58	(3%)	298	(44%)	2,235	(43%)
Appropriate cord care (CordCare)	3,261	(77%)	78	(4%)	155	(23%)	548	(11%)
Clean instrument used to cut cord (CordCut)	3,253	(77%)	742	(41%)	393	(58%)	3,610	(70%)
Wrapped within 5 mins (BabyWarm)	3,099	(73%)	88	(5%)	0	(0%)	352	(7%)
Bathing after 6h (Bathing)	3,149	(74%)	120	(7%)	0	(0%)	372	(7%)
Breast feeding Initiated within 1h (Breast)	411	(10%)	86	(5%)	13	(2%)	374	(7%)
No prelacteal food (OnlyBreastMilk)	375	(9%)	79	(4%)	0	(0%)	326	(6%)
Postnatal visit on 1st day (PNCChild_Day)	51	(1%)	3	(0%)	0	(0%)	11	(0%)
