# Supplementary material for: Socioeconomic inequalities in newborn care during facility and home deliveries: a cross sectional analysis of data from demographic surveillance sites in rural Bangladesh, India and Nepal
Source: Int J Equity Health. 2018 Aug 15;17:119. doi: 10.1186/s12939-018-0834-9 (PMC6094873; doi:10.1186/s12939-018-0834-9)
Supplement: Supplementary file 2 — Table S5. Complete case analysis: The distribution (%) of literacy, delivery type and delivery type by literacy in the population of the study sites. Table S6. Complete case analysis: A univariable model of the effect of literacy on newborn care score. Table S7. Complete case analysis: A multivariable model of the effect of literacy and delivery type on newborn care score including interaction. (PDF 86 kb) [file 12939_2018_834_MOESM2_ESM.pdf]

Appendix 3

Table 3. Complete case analysis: The distribution (%) of literacy, delivery type and delivery type by literacy in the population of the study sites.

	Ekjut	PCP	Dhanusha	Makwanpur
Number of deliveries	8720	26835	17835	6688
Literacy				
Illiterate (%)	68%	34%	76%	66%
Literate (%)	32%	66%	24%	34%
Delivery Type				
Home (%)	78%	80%	78%	97%
Home + SBA (%)	4%	3%	1%	0%
Institutional (%)	19%	17%	21%	2%
Delivery type by literacy				
Literate				
Home (%)	61%	76%	63%	95%
Home + SBA (%)	5%	3%	1%	1%
Institutional (%)	34%	21%	35%	5%
Illiterate				
Home (%)	85%	89%	83%	99%
Home + SBA (%)	3%	2%	1%	0%
Institutional (%)	12%	8%	16%	1%
OR facility delivery literate vs. Illiterate (95% CI)	3.94 (3.49 - 4.45)	3.05 (2.80 - 3.32)	2.89 (2.65 - 3.16)	4.31 (3.50 - 5.32)
OR home with SBA literate vs. Illiterate (95% CI)	2.73 (2.16 - 3.44)	1.46 (1.27 - 1.68)	2.28 (1.74 - 2.98)	3.38 (1.85 - 6.18)

Appendix 3

Table 5. Complete case analysis: A univariable model of the effect of literacy on newborn care score.

	India		*Bangladesh*		*Nepal (Dhanusha)*		*Nepal (Makwanpur)*	
	beta	*(95% CI)*	*beta*	*(95% CI)*	*beta*	*(95% CI)*	*beta*	*(95% CI)*
Literate	0.59	(0.53 - 0.66)	0.29	(0.25 - 0.33)	0.51	(0.44 - 0.59)	0.45	(0.30 - 0.59)
Constant (illiterate)	3.77	(3.52 - 4.02)	4.86	(4.54 - 5.17)	3.20	(3.03 - 3.37)	3.91	(3.67 - 4.15)

Appendix 3

Table 7. Complete case analysis: A multivariable model of the effect of literacy and delivery type on newborn care score including interaction.

	India		Bangladesh		Nepal (Dhanusha)		Nepal (Makwanpur)	
	beta	(95% CI)	beta	(95% CI)	beta	(95% CI)	beta	(95% CI)
Literate	0.27	(0.20 - 0.35)	0.22	(0.18 - 0.26)	0.41	(0.34 - 0.49)	0.44	(0.30 - 0.58)
Home + SBA	1.06	(0.85 - 1.26)	0.65	(0.49 - 0.82)	1.13	(0.86 - 1.39)	0.87	(0.04 - 1.70)
Institutional	1.12	(1.00 - 1.23)	1.63	(1.45 - 1.80)	1.72	(1.55 - 1.89)	2.36	(1.96 - 2.77)
Home + SBA*literate	0.21	(-0.07 - 0.50)	0.01	(-0.20 - 0.21)	0.05	(-0.37 - 0.46)	1.04	(0.03 - 2.05)
Institutional*literate	0.34	(0.18 - 0.51)	-0.11	(-0.31 - 0.08)	-0.07	(-0.33 - 0.19)	-0.60	(-1.04 - -0.17)
Constant (Home-SBA, illiterat	3.63	(3.38 - 3.87)	4.77	(4.47 - 5.08)	3.09	(2.92 - 3.26)	3.76	(3.56 - 3.97)
